# Pleomorphic Adenoma of the External Auditory Canal: A Rare Presentation

**DOI:** 10.1155/2015/696531

**Published:** 2015-05-27

**Authors:** Samir Jaber, Milan Rudic, Ivan James Keogh

**Affiliations:** ^1^Department of Otorhinolaryngology, University Hospital Galway, ENT OPD, Galway, Ireland; ^2^Discipline of Otorhinolaryngology, National University of Ireland Galway, Galway, Ireland

## Abstract

A 55-year-old male presented with a nine-month history of gradually enlarging, painless mass in the right external auditory canal associated with hearing loss and occasional bleeding. Examination demonstrated complete obstruction of the outer 1/3 of the external auditory canal by a firm, pink, rubbery mass. CT scan of the temporal bone showed tumor mass with no evidence of bone destruction. The tumor was excised and histopathology confirmed a diagnosis of ceruminous pleomorphic adenoma of the external auditory canal. Six months following the surgery, patient is free of any recurrent disease.

## 1. Introduction

Pleomorphic adenoma (PA) is the most common benign tumor of the parotid glands, accounting for 80% of all parotid neoplasms [1]. It is a slowly growing tumor [2], lobular, not well encapsulated, and with recurrence rate of 1–5% after appropriate excision [1]. PA of the external auditory canal (EAC) is extremely rare and belongs to a group of benign and malignant tumors of the EAC, derived from the ceruminous glands [3]. Approximately 5% of all external ear neoplasms are benign adenomas [4]. We report a case of pleomorphic adenoma of the external auditory canal in a 55-year-old male patient and discuss its clinical presentation, diagnosis, and treatment options.

## 2. Case Report

**Figure 1:**
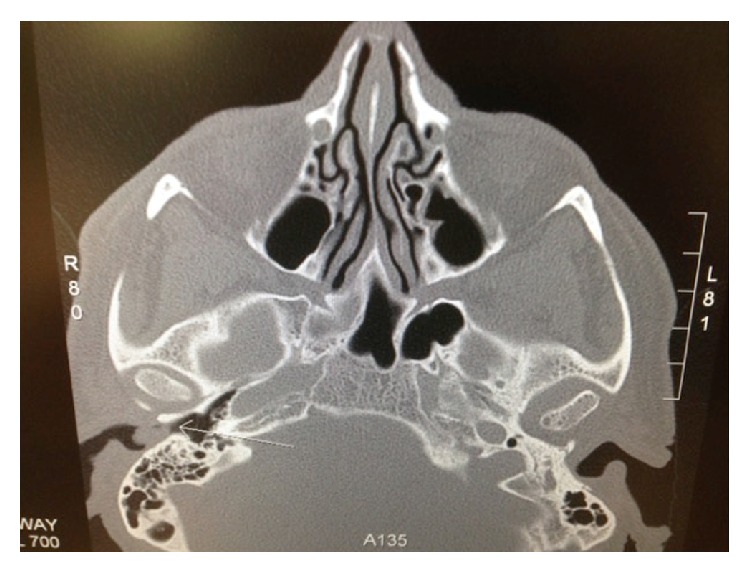
CT scan of the temporal bones, axial view, showing 1 cm polypoid soft tissue mass (arrow) in the external auditory canal, with no underlying exostosis or osteoma identified.

**Figure 2:**
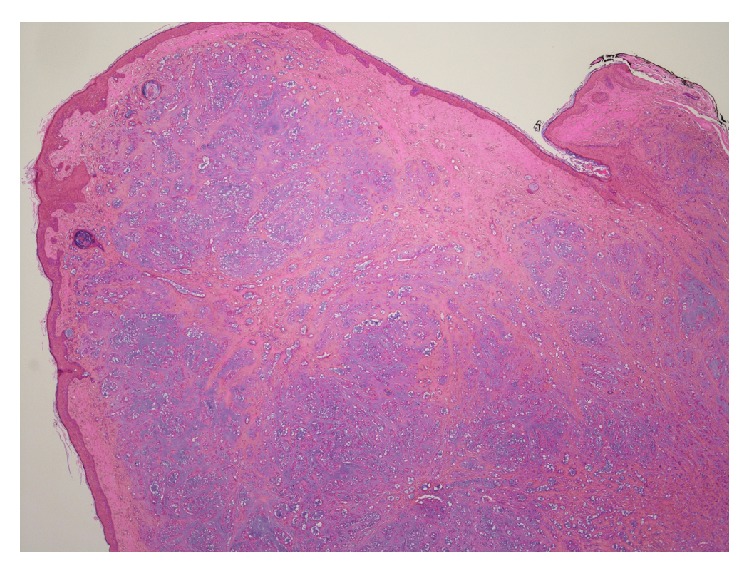
Histology showing polypoidal lesion covered by squamous mucosa (H&E, ×100).

**Figure 3:**
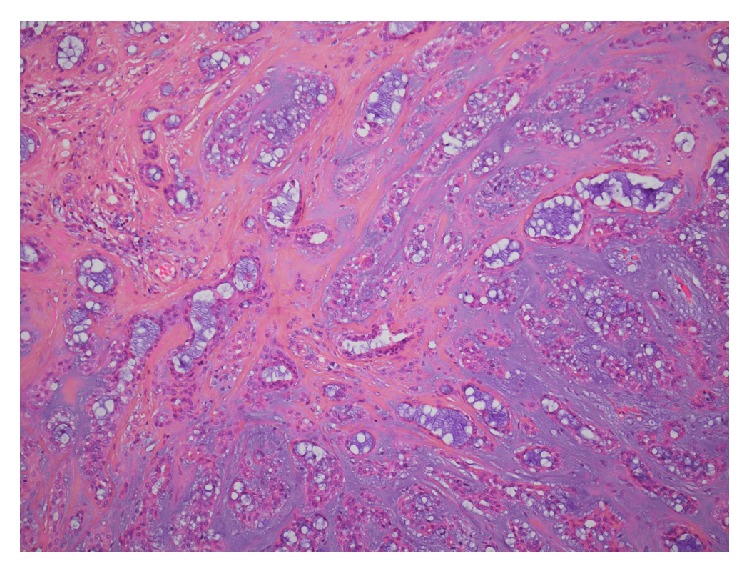
Histology showing variably sized glands, embedded into myxofibrous stroma. The glands show some mucin production (H&E, ×200).

A 55-year-old male patient was seen in ENT outpatients with a growth in the right external auditory canal. The growth was painless but increased in size over a period of nine months, causing hearing loss and occasional bleeding from the canal. On examination the patient had a pink, rubbery mass obstructing the lateral 1/3 of the external auditory canal. Tympanic membrane was intact and other ENT findings were normal. The patient underwent a CT scan of the temporal bone that showed 1 cm polypoid soft tissue growth in the external auditory canal with no underlying exostosis, osteoma, or erosion (Figure 1). Middle and inner ear structures appeared normal. Under general anesthesia the patient underwent a wide local excision of the tumor via a retroauricular approach. The tumor was attached to the skin of the external auditory canal and was removed with overlying skin. Exposed bone was covered with split thickness skin grafts and supported with silastic tubing and BIPP gauze dressings. Postoperative recovery was uneventful. Histopathology confirmed the tumor as a polypoid lesion covered by squamous mucosa, with variably sized mucin producing glands, embedded in myxofibrous stroma (Figure 2). The mucins producing glands demonstrated no features of any atypia or mitotic activity and were lined by cuboidal cells (Figure 3). Further immunohistochemistry demonstrated strong immunoreactivity of the glands in keeping with glandular/ductal differentiation. The final diagnosis was pleomorphic adenoma of the external auditory canal. Six months following the surgery there is no sign of any tumor recurrence and external auditory canal is fully healed.

## 3. Discussion

Pleomorphic adenomas are the most common salivary gland tumors [5]. Primary site is the parotid gland, 85% [1, 6, 7]. Though pleomorphic adenomas of the external auditory canal are extremely rare, they can arise from the ceruminous glands, which are located in the outer 1/3 of the external auditory canal [8, 9]. They can also arise from ectopic salivary gland tissue or extension of parotid pleomorphic adenoma into the ear through foramen of Huschke [10, 11]. The external auditory canal consists of fibrous stroma covered by squamous epithelium. The outer one-third of the auditory canal contains ceruminous and sebaceous glands, which are absent in the inner two-thirds of the ear canal and the middle ear. Primary tumors of the external auditory meatus are very rare; they grow slowly and affect both sexes equally with median age of 50 years [12]. Five percent of these tumors are ceruminous (modified sweat) gland tumors [3]. Benign ceruminous gland tumors include adenoma, chondroid syringoma (pleomorphic adenoma), and syringocystadenoma papilliferum, while malignant ceruminous gland tumors include adenocarcinoma, adenoid cystic carcinoma, and mucoepidermoid carcinoma [10, 13]. Chondroid syringoma is the term that describes pleomorphic adenoma arising from skin appendages. They are characterized by the presence of subepithelial proliferation of glandular structures with nets of myoepithelial components in a chondromyxoid stroma [14, 15]. Myoepithelial cells of the ceruminous glands are precursor of primary pleomorphic adenoma of the external ear canal. Cerumen pigment and immunohistochemistry with CK7 can help to distinguish this tumor from other neoplasms [10]. Although transformation of these tumors into malignancy is rare, there is a reported case of pleomorphic adenoma of the external auditory canal alteration into aggressive chondroid syringoma [15, 16]. These tumors should be considered as part of the differential diagnosis of any painless mass of the external auditory canal.

Current management of pleomorphic adenoma of the external auditory canal is complete surgical excision with adequate margins [17]. Recurrence is rare; however long-term follow-up is recommended [11].

In this case the tumor was completely excised surgically, and six months post-op there are no signs of recurrence.
